# Efficacy and Safety of Tenecteplase in Acute Ischemic Stroke: A Meta‐Analysis of Randomized Controlled Trials

**DOI:** 10.1002/brb3.70791

**Published:** 2025-09-01

**Authors:** Zaryab Bacha, Javeria Javed, Maheen Sheraz, Muhammad Abdullah Ali, M. Ehtisham Wali Khan, Sufyan Shahid, Asad Iqbal, Mahnoor Qasim, Abdullah Afridi, Fazia Khattak, Fathimathul Henna, Mazhar Ali Shah, Raheel Ahmed

**Affiliations:** ^1^ Khyber Medical College Peshawar Pakistan; ^2^ Jinnah Sindh Medical University Karachi Pakistan; ^3^ Continental Medical College Lahore Pakistan; ^4^ Khawaja Muhammad Safdar Medical College Sialkot Pakistan; ^5^ Bacha Khan Medical College Mardan Pakistan; ^6^ Dubai Medical College for Girls Dubai UAE; ^7^ National Heart and Lung Institute, Imperial College London London UK

**Keywords:** acute ischemic stroke (AIS), hemorrhage, recanalization, tenecteplase (TNK), thrombolysis

## Abstract

**Introduction:**

Acute ischemic stroke (AIS) is the most common type of stroke, with increasing incidence and significant healthcare costs. Tenecteplase (TNK), a modified variant of tissue plasminogen activator (tPA), offers advantages such as a longer half‐life and single‐bolus administration. This meta‐analysis evaluates the safety and efficacy of TNK compared to non‐thrombolytic management in AIS to guide clinical decision‐making.

**Methodology:**

A comprehensive literature search across major databases identified randomized controlled trials (RCTs) comparing tenecteplase with non‐thrombolytic care in ischemic stroke. Data extraction and bias assessment were conducted independently, using RoB 2.0 and the GRADE framework. Meta‐analysis was performed using RevMan 5.4.1, applying random‐effects models and assessing heterogeneity with the *I* ^2^ statistic.

**Results:**

This meta‐analysis included seven studies with 3266 patients and found no significant difference between tenecteplase and standard medical care in terms of the mRS score at 90 days (mean difference = −0.16, *p* = 0.58), functional independence (mRS 0–2 at 90 days) (odds ratio = 1.07, *p* = 0.51), and reperfusion (TICI 2b‐3 at 24 h) (odds ratio = 1.33, *p* = 0.39). However, tenecteplase was associated with significantly higher mRS 0–1 at 90 days (odds ratio = 1.22, *p* = 0.01), better recanalization at 24 h (odds ratio = 3.28, *p* = 0.04), and improved NIHSS scores at 7 days (mean difference = −0.71, *p* = 0.003). On the downside, tenecteplase showed a significantly higher incidence of symptomatic intracranial hemorrhage (SICH) within 36 h (odds ratio = 2.24, *p* = 0.04) and any ICH (odds ratio = 1.40, *p* = 0.04), with no significant differences in mortality at 90 days (odds ratio = 1.18, *p* = 0.33) or stroke recurrence (odds ratio = 1.23, *p* = 0.55) and Barthel Index Score (odds ratio = 1.09, *p* = 0.69) and quality of life. Serious adverse events were slightly higher in the tenecteplase group but did not reach statistical significance (odds ratio = 1.18, *p* = 0.23).

**Conclusion:**

Tenecteplase improves early neurological recovery and recanalization and provides excellent functional outcomes in acute ischemic stroke. However, it is associated with a higher risk of symptomatic and overall intracranial hemorrhage. Mortality, stroke recurrence, and overall functional independence remain unaffected.

## Graphical abstract

This meta‐analysis of randomized controlled trials evaluated tenecteplase versus standard treatment in acute ischemic stroke. Tenecteplase enhanced recanalization and NIHSS scores but showed higher early symptomatic intracranial hemorrhage. No notable differences were seen in mortality or functional independence, supporting cautious use in selected patients.

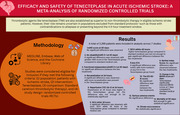

## 1 Introduction

Acute ischemic stroke (AIS) accounts for a significant 68.16 million cases globally out of 89.13 million cases, making it one of the most prevalent type of stroke (Capirossi et al. 2023). The inclining trend in AIS cases is associated with a huge financial burden as well. From 2015 to 2035, it is estimated that total direct medical costs related to stroke will double, escalating from $36.7 billion to $94.3 billion (Capirossi et al. 2023). AIS occurs when the brain's blood supply is interrupted, resulting in severe damage to the brain cells (Walter 2022). As a medical emergency, AIS demands immediate and effective intervention to minimize brain damage and improve recovery outcomes (Walter 2022).

For several years and continuing to the present, the gold standard and the United States Food and Drug Administration (FDA)‐approved (Meng et al. 2024) intervention for acute ischemic stroke has been the use of thrombolytic therapy, specifically tissue plasminogen activator (tPA), one of its type is known as alteplase (Baig and Bodle 2023; Reed et al. 2023). Alteplase is administered at a dose of 0.9 mg/kg, with 10% as an IV bolus, while remaining is given over the time period of 60 min, within 4.5 h of symptom onset. It offers a favorable benefit–risk ratio, particularly for patients who are older than 80. However, a short therapeutic window and the possibility of hemorrhagic consequences limit its utilization.

In addition, non‐thrombolytic therapy options for managing AIS included approaches such as endovascular thrombectomy, antiplatelet therapy, and supportive care. According to the American College of Chest Physicians, aspirin therapy, which is a form of anti‐platelet therapy, can be used in patients with AIS within 48 h to significantly reduce the risk of early recurrent ischemic stroke, provided there is no high risk of early hemorrhagic complications. However, the disadvantages of this therapy include twice‐daily dosing and headaches, while endovascular thrombectomy is associated with poor functional outcomes and a high mortality rate (Bluhmki et al. 2020; Whiteley et al. 2016).

Tenecteplase (TNK), a genetically modified variant of the tissue plasminogen activator alteplase, has emerged as a potential alternative (Sandercock et al. 2008). The characteristics of TNK includes improved fibrin specificity which leads to longer half‐life and ease of administration (Sandercock et al. 2008). Several meta‐analyses have compared TNK with other thrombolytic agents, such as alteplase (Elfil et al. 2025; Hackam and Spence 2019; Lansberg et al. 2012). These studies consistently concluded TNK being non‐inferior to alteplase (Elfil et al. 2025; Hackam and Spence 2019; Lansberg et al. 2012). Specifically, TNK has been shown to achieve excellent functional outcomes at 90 days without increasing the risk of safety concern and reduced disability at 3 months, such as bleeding (Hackam and Spence 2019; Lansberg et al. 2012). Thrombolytic agents such as TNK are also established as superior to non‐thrombolytic therapy in eligible ischemic stroke patients. However, their role remains uncertain in populations excluded from standard protocols—such as those with contraindications to alteplase or presenting beyond the 4.5‐h treatment window.

Prior meta‐analyses, such as that by Palaiodimou et al. (2024b), were limited by incomplete evidence, as key trials such as TIMELESS were only available in preliminary form, and landmark studies such as ATTENTION‐IA, TEMPO‐2, and POST‐TNK had not yet been published. These newer studies directly compare TNK to medical therapy in patients outside alteplase criteria or time windows. To address this evolving landscape and help clinicians make informed decisions for patients ineligible for alteplase, we conducted an updated and comprehensive meta‐analysis comparing TNK with standard (non‐thrombolytic) medical therapy. By incorporating all available randomized trials to date, our study aims to provide a more conclusive assessment of TNK's efficacy and safety in this specific clinical context.

## 2 Methods

This systematic review and meta‐analysis was conducted in accordance with the Preferred Reporting Items for Systematic Reviews and Meta‐Analyses (PRISMA) guidelines (Page et al. 2021) with the study protocol prospectively registered on PROSPERO (CRD420251025979).

### 2.1 Search Strategy

A comprehensive search was conducted across major databases, including MEDLINE, Embase, Web of Science, and the Cochrane Library, covering all available literature from inception up to March 30, 2025, without any language restriction. We conducted a comprehensive search using the terms “Tenecteplase” [MeSH] OR “Metalyse” OR “TNKase” in combination with “Ischemic Stroke” [MeSH] OR “Ischemic Strokes” OR “Stroke, Ischemic” OR “Ischaemic Stroke” OR “Ischaemic Strokes” OR “Stroke, Ischaemic” OR “Acute Ischemic Stroke” OR “Acute Ischemic Strokes” OR “Ischemic Stroke, Acute” OR “Stroke, Acute Ischemic” OR “Cryptogenic Ischemic Stroke” OR “Cryptogenic Ischemic Strokes”, to identify relevant studies evaluating the use of TNK in the management of ischemic stroke. Gray literature, including dissertations and unpublished research, was not included in this review. The complete search strategy is detailed in Table S1.

### 2.2 Eligibility Criteria and Study Selection

Studies were considered eligible for inclusion if they met the following criteria: (1) population: patients with ischemic stroke, (2) intervention: TNK, (3) comparison: standard care (non‐thrombolytic therapy), and (4) study design: randomized controlled trials (RCTs).

The inclusion criteria were limited to RCTs that directly compared TNK with non‐thrombolytic agents in patients diagnosed with ischemic stroke who were ineligible for standard thrombolysis (e.g., alteplase) or presented outside the conventional treatment window. All other study types, including observational studies, case reports, and non‐randomized trials, were excluded. Additionally, studies comparing TNK with other thrombolytic agents or those lacking a non‐thrombolytic control group, as well as studies not focusing on ischemic stroke patients or lacking sufficient data, were excluded from the review.

After duplicate removal using Rayyan, two investigators (M.A.A. and E.W.) independently screened titles and abstracts based on the eligibility criteria. Any disagreements were first resolved through discussion and consensus. In cases where consensus was not achieved, a third reviewer (S.S.) was consulted to make the final decision. Full texts of the selected articles were then reviewed to confirm their inclusion in both qualitative and quantitative analyses. Any disagreements were resolved through consultation with a third author (S.S.).

### 2.3 Data Extraction

Two authors (M.A.A. and S.S.) independently extracted data using a predesigned Microsoft Excel spreadsheet. Any disagreements during the process were resolved by a third author (Z.B.). Data were collected from the main text, tables, and figures of the included studies, with raw values estimated from reported percentages when required. Extracted data included study characteristics, baseline characteristics, and outcomes. Study characteristics included author name, publication year, follow‐up time, and outcomes reported, while baseline characteristics included such as study id, country, sample size, mean age, gender, hypertension, diabetes, smoking, previous stroke and atrial fibrillation.

Outcomes were categorized into primary outcomes which included modified Rankin scale (mRS) at 90 days, mRS 0–1 at 90 days, mRS 0–2 at 90 days, while secondary outcomes included recanalization at 24 h, reperfusion at 24 h, symptomatic intracranial hemorrhage at 36 h, symptomatic intracranial hemorrhage at 36 h, any intracranial hemorrhage, Barthel index score >95, change in the NIHSS score at 90 days, EQ‐5D‐5L (quality of life) at 90 days, mortality at 90 days, serious adverse events, and stroke recurrence.

### 2.4 Bias Evaluation

To evaluate the risk of bias, the Cochrane Risk‐of‐Bias tool for randomized trials (RoB 2.0) was used (Sterne et al. 2019). This tool assesses risk of bias across five domains: (1) bias due to randomization process, (2) bias due to deviation from the intended intervention, (3) bias due to missing outcome data, (4) bias due to measurement of outcome, and (5) bias due to selection of reported results. Bias in all studies was rated as high, low, and some concerns.

### 2.5 Grade Assessment

The quality of the evidence for this meta‐analysis was assessed by two independent reviewers (A.K. and M.A.A.) using the GRADE framework, with the GRADEpro Guideline Development Tool (Santesso et al. 2016). The evidence was classified into levels ranging from high to very low (Shao et al. 2023). Any discrepancies in the assessment were resolved through mutual agreement.

### 2.6 Statistical Analysis

All meta‐analyses were performed using Review Manager (RevMan) version 5.4.1. For dichotomous outcomes, risk ratios (RR) with 95% confidence intervals (CI) were calculated using the Mantel–Haenzel method, while for continuous outcomes, mean differences (MD) with 95% CI were determined using the inverse variance method. Random‐effects meta‐analyses were applied throughout, and the results were displayed in forest plots. The Higgins *I* ^2^ statistic was used to assess the heterogeneity of the included studies. An *I* ^2^ value between 0% and 50% was considered indicative of low heterogeneity, whereas a value above 50% was taken to suggest significant heterogeneity. In cases where *I* ^2^ exceeded 50%, a sensitivity analysis was performed to address the variability.

### 2.7 Subgroup Analysis

Subgroup analysis was performed for possible variables if any.

## 3 Results

### 3.1 Search Results

**FIGURE 1:**
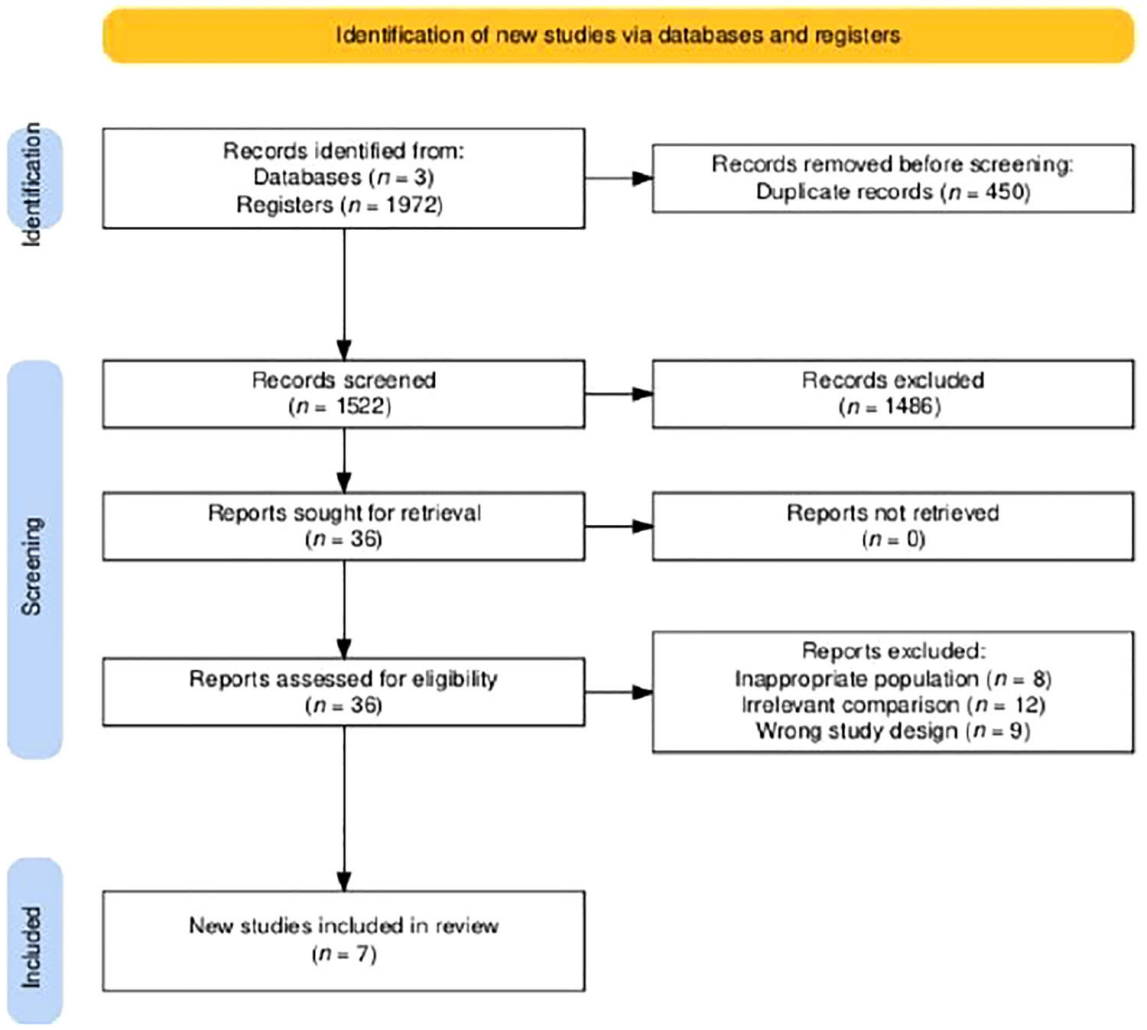
PRISMA flowchart of screening process.

A total of 1975 records were identified from databases (*n* = 3). After removing 450 duplicate records, 1522 records were screened, out of which 1487 were excluded. Thirty‐five reports were sought for retrieval, and all were successfully retrieved. After assessing 35 reports for eligibility, 29 were excluded due to inappropriate population (*n* = 8), irrelevant comparison (*n* = 12), and wrong study design (*n* = 9). Finally, seven studies were included in the review (Figure 1).

### 3.2 Study Characteristics

A total of 3266 patients were included in analysis across seven studies (Albers et al. 2024; Coutts et al. 2024; Hu et al. 2025; Huang et al. 2025; Roaldsen et al. 2023; Xiong et al. 2024; Wang et al. 2023). The studies were published in China, Norway, Denmark, Switzerland, Lithuania, the United States, and Canada. Publication years ranged from 2023 to 2025 (Table 1). Table 2 provides the summary of baseline characteristics for the included studies.

**TABLE 1:** Study characteristics.

Study ID	Year		Country	Intervention	Control	0utcomes	Follow‐up
ROSE‐TNK	2023		China	Tenecteplase	Standard medical therapy	Primary: mRS score of 0 or 1 at 90 days; secondary: mRS score of 0–2 at 90 days, change in the NIHSS score from baseline at 5–7 days or discharge if earlier, death within 90 days, symptomatic intracranial hemorrhage within 48 h; serious adverse event	90 days
TWIST trial	2023		Norway, Denmark, Switzerland, Lithuania	Tenecteplase	Standard medical therapy	Primary: functional improvement in the mRS at 90 days (scale 0–6); secondary: excellent functional outcomes at 90 days, good functional outcome, response to treatment according to baseline neurological deficit; safety: death within 90 days after intervention, symptomatic intracranial hemorrhage, parenchymal hemorrhage, any intracranial hemorrhage, major extracranial bleeding	90 days
ATTENTION‐IA trial	2024		China	Tenecteplase	Standard medical therapy	Primary: mRS score 0–1 at 90; secondary: median mRS distribution, mRS score 0–2 at 90 days, mRS score 0–3 at 90 days, median NIHSS score 24–72 h, median NIHSS score at 5–7 days or discharge, Barthel index score of 95 or 100 at 90 days, median EQ‐5D‐5L score at 90 days; safety: all‐cause mortality ≥90 days, all‐cause mortality ≥7 days, symptomatic ICH ≥36 h; secondary imaging efficacy and safety: patency at 24–72 h on CTA/MRA, radiological intracranial hemorrhage 36≥ h, asymptomatic ICH	90 days
TRACE‐III trial	2024		China	Tenecteplase	Standard medical therapy	Primary: score of 0 or 1 on the modified Rankin scale at 90 days; secondary: ordinal distribution of scores on the modified Rankin scale at 90 days, score of ≥2 on the modified Rankin scale, indicating functional independence at 90 days, major neurologic improvement at 72 h, reperfusion at 24 h, change in the NIHSS score at 7 days; safety: symptomatic intracranial hemorrhage within 36 h after randomization, death within 90 days, moderate or severe systemic bleeding within 90 days, any adverse event, any serious event	NR
TIMELESS trial	2024		United States	Tenecteplase	Standard medical therapy	Primary: median score on the modified Rankin scale at 90 days; secondary: functional independence at 90 days, recanalization at 24 h, reperfusion at 24 h, reperfusion at the conclusion of endovascular thrombectomy; safety: death, symptomatic intracranial hemorrhage within 36 h, parenchymal hematoma within 72 h	90days
TEMPO‐2 trial	2024		Canada	Tenecteplase	Standard medical therapy	Primary: responder; secondary: mRS 0–1 at 90 days, mRS at 90 days, NIHSS of 0 at 5 days or discharge, mRS return to pre‐morbid function, mean mRS score at 90 days, Lawton IADL percent functioning score, EQ‐5D‐5L index score, EQ‐5D‐5L VAS score and death at 90 days	90days
POST‐TNK trial	2025		China	Tenecteplase	Standard medical therapy	Primary: mRS score of 0 or 1 at 90 days; secondary: mRS score of 0–2 at 90 days, mRS score at 90 days, No. of wins/total No. of pairs, mRS score at 90 days, change in the NIHSS score from baseline at 5–7 days or discharge if earlier, EQ‐5D‐5L score at 90 days, No. of wins/total No. of pairs, EQ‐5D‐5L score at 90 days; primary safety: death within 90 days, symptomatic intracranial hemorrhage within 48 h; secondary safety: any radiologic intracranial hemorrhage within 48 h, systemic bleeding	90 days

**TABLE 2:** Baseline characteristics of included studies.

Study ID	Sample size	Age (years)‐mean (SD)	Male‐*n* (%)	Diabetes‐*n* (%)	HTN‐*n* (%)	Current smoker‐*n* (%)	Previous stroke‐*n* (%)	Atrial fibrillation‐*n* (%)
ROSE‐TNK 2023	80	62.68 (8.87)/62.80 (8.56)	31 (77.5)/26 (65.0)	9 (22.5)/13 (32.5)	24 (60.0)/28 (70.0)	19 (47.5)/21 (52.5)	11 (27.5)/12 (30.0)	NR
TWIST trial‐2023	578	73.7 (10.73) vs. 73.7 (12.07)	164 (57) vs. 168 (58)	55/278 (20) vs. 52/281 (19)	176/276 (64) vs. 177/279 (63)	51/240 (21) vs. 46/229 (20)	75/277 (27) vs. 60/274 (22)	55/267 (21) vs. 31/272 (11)
ATTENTION‐IA trial‐2024	208	65 (11.3) vs. 67.3 (10.8)	84 (80.8) vs. 73 (70.2)	29 (27.9) vs. 29 (27.9)	73 (70.2) vs. 88 (84.6)	23 (22.1) vs. 27 (26)	NR	21 (20.2) vs. 30 (28.9)
TRACE‐III trial‐2024	516	66.67(12.67) vs. 67.67(12.68)	183 (69.3) vs. 167 (66.3)	69 (26.1) vs. 71 (28.2)	177 (67) vs. 180 (71.4)	NR	NR	49 (18.6) vs. 48 (19)
TIMELESS trial‐2024	458	73.33 (7.46) vs. 72.67 (14.17)	73.33 (7.46) vs. 72.67 (14.17)	106 (46.49) vs. 107 (46.5)	NR	NR	NR	NR
TEMPO‐2 trial‐2024	886	70.67 (13.39) vs. 71.33 (13.39)	244 (56) vs. 272 (60)	82(19) vs. 86 (19)	265 (61) vs. 261 (58)	NR	72 (17) vs. 85 (19)	91 (21) vs. 78 (17)
POST‐TNK trial‐2025	540	68 (12.67) vs. 68 (12.67)	154 (57.2) vs. 165 (60.9)	45 (16.7) vs. 56 (20.7)	145 (53.9) vs. 156 (57.6)	80 (30.9) vs. 78 (28.8)	55 (20.4) vs. 44 (16.2)	126 (46.8) vs. 124 (45.8)

### 3.3 Quality Assessment

Seven RCTs demonstrated a low risk of bias, while one trial raised some concerns regarding deviations from the intended intervention. A detailed assessment of the risk of bias for each study is provided in Figure S1.

### 3.4 Certainty of Evidence

The GRADE approach, using the GRADE pro Guideline Development Tool, was employed to assess the certainty of evidence. A detailed assessment is shown in Table S2.

### 3.5 Meta Analysis of Primary Outcomes

#### 3.5.1 mRS Score at 90 Days

No significant difference was observed between the TNK group and the standard medical care group (mean difference = −0.16, 95% CI: −0.74, 0.41; *p* = 0.58, Figure 04(A)). Heterogeneity between the studies was high (*I* ^2^ = 85%). After omitting ATTENTION‐IA trial on leave‐one‐out analysis, heterogeneity dropped to 0% (Figure S2).

#### 3.5.2 No Disability (mRS 0–1 at 90 days)

A significant difference was observed, with a greater mRS score in the TNK group compared to the standard medical care group (odds ratio = 1.22, 95% CI: 1.04, 1.42; *p* = 0.01, Figures 02(A) and 4(B). Heterogeneity between the studies was low (*I* ^2^ = 6%).

#### 3.5.3 Functional Independence (mRS 0–2 at 90 days)

**FIGURE 2:**
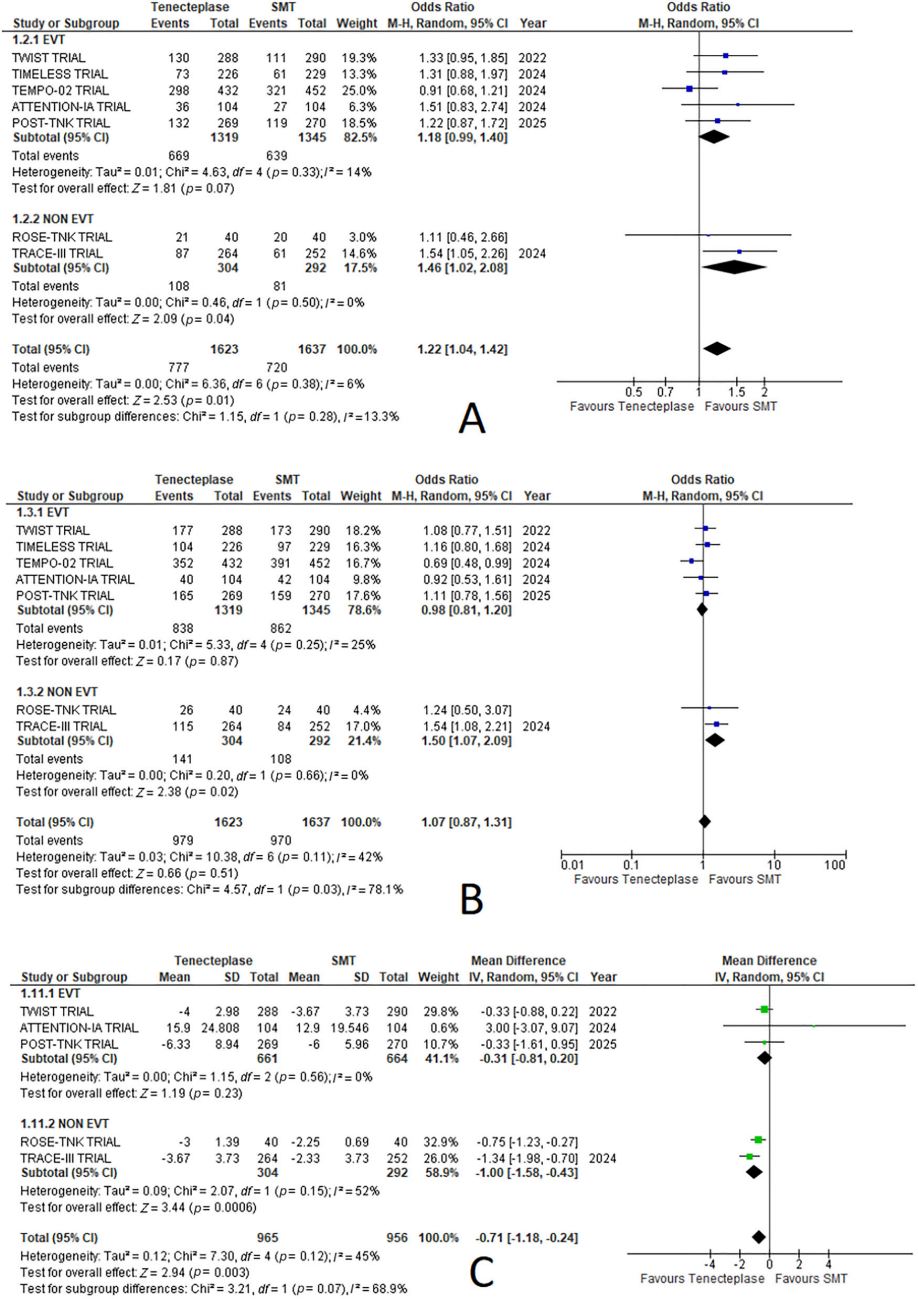
Forest plots for EVT‐based subgroup analysis of outcomes comparing tenecteplase with standard medical treatment (SMT): (A) no disability (mRS 0–1 at 90 days), (B) functional independence (mRS 0–2 at 90 days), and (C) change in the NIHSS score at 7 days.

**FIGURE 3:**
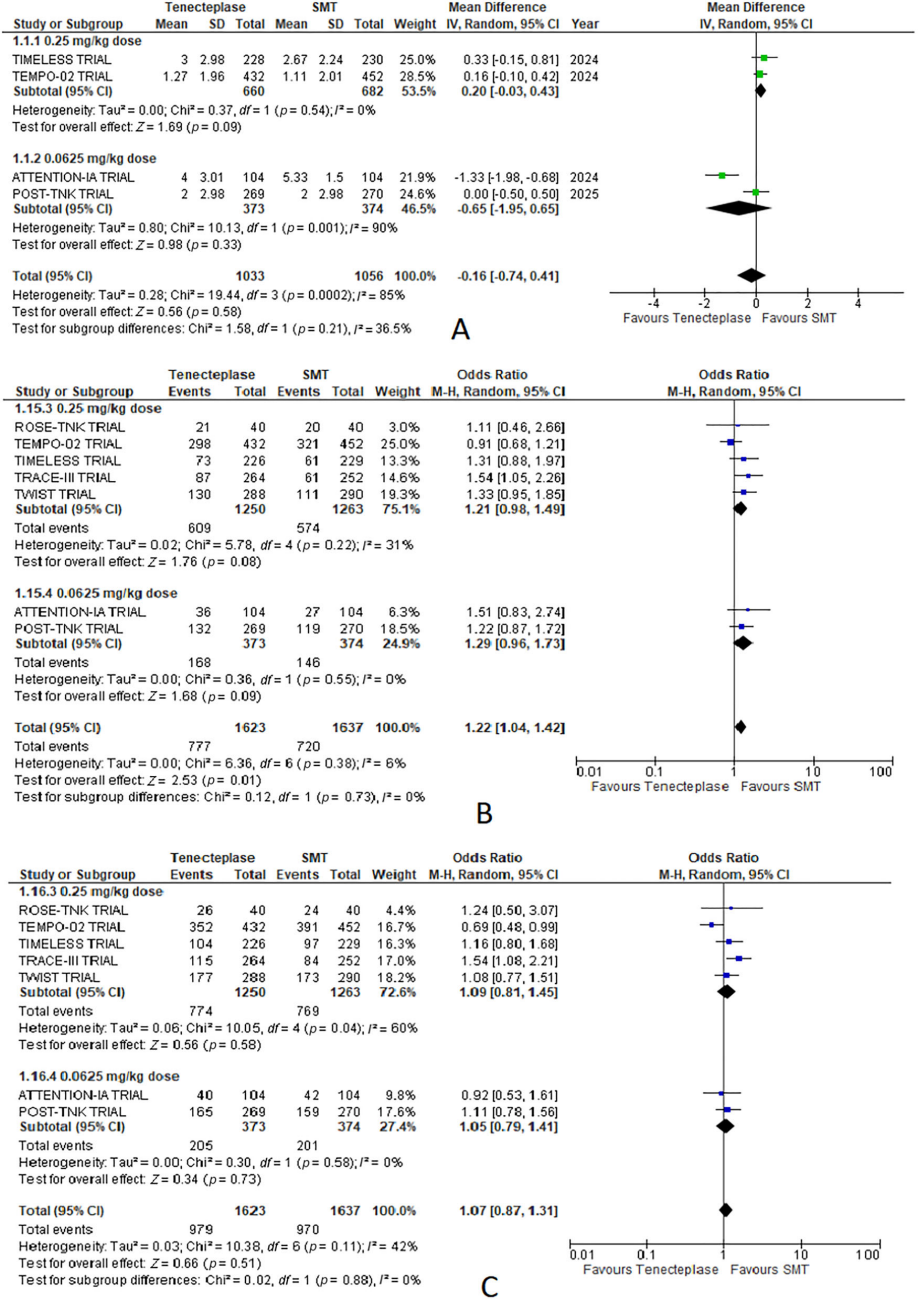
Forest plots of dosed‐based subgroup analysis of outcomes comparing tenecteplase with standard medical treatment (SMT): (A) mean mRS score at 90 days, (B) no disability(mRS 0–1 at 90 days), and (C) functional independence (mRS 0–2 at 90 days).

Functional independence (mRS 0–2 at 90 days) was comparable between both groups with no significant difference between the two treatments (odds ratio = 1.07, 95% CI: 0.87, 1.31; *p* = 0.51, Figure 02(C)). Heterogeneity between the studies was moderate (*I* ^2^ = 42%) (Figures 2B and 3C).

### 3.6 Meta Analysis of Secondary Outcomes

#### 3.6.1 Recanalization at 24 Hours

**FIGURE 4:**
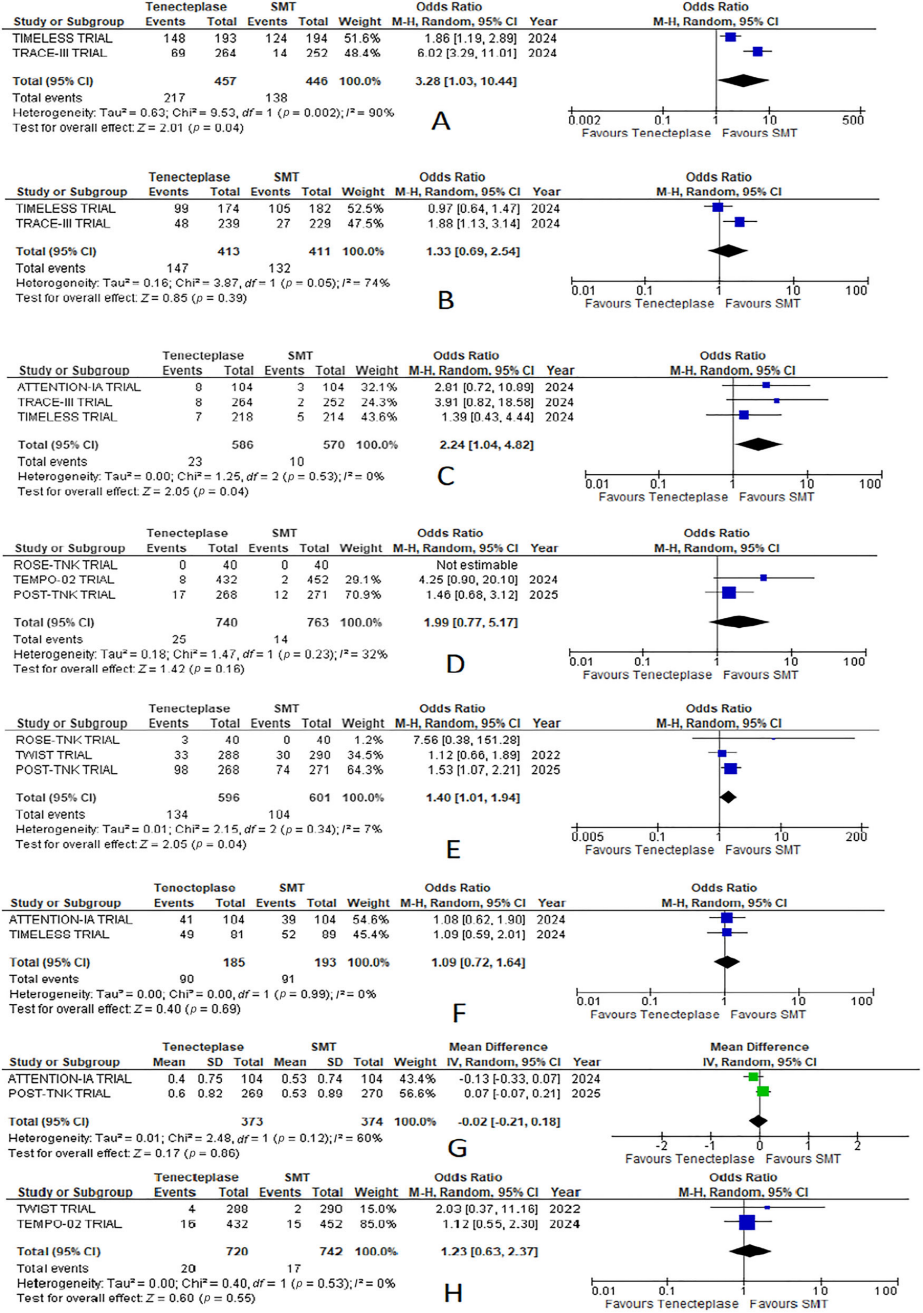
Forest plots of secondary outcomes comparing tenecteplase with standard medical treatment (SMT): (A) recanalization at 24 h, (B) reperfusion (TICI 2b‐3) at 24 h, (C) SICH within 36 h, (D) SICH within 48 h, (E) any ICH, (F) Barthel index score >95, (G) EQ‐5D‐5L (quality of life) at 90 days, and (H) stroke recurrence.

A significant difference was observed, with more recanalization was present in the TNK group (odds ratio = 3.28, 95% CI: 1.03, 10.44; *p* = 0.04, Figure 03(A)). High heterogeneity was present between the studies (*I* ^2^ = 90%). No sensitivity and subgroup analysis was done for this outcome as only two studies reported it (Figure 4A).

#### 3.6.2 Reperfusion (TICI 2b‐3) at 24 h

Reperfusion at 24 h was higher in the TNK group, but the difference was not enough to be statistically significant (odds ratio = 1.33, 95% CI: 0.69, 2.54; *p* = 0.39, Figure 03(B)). Heterogeneity between the studies was moderate (*I* ^2^ = 74%). No sensitivity and subgroup analysis was done for this outcome as only two studies reported it (Figure 4B).

#### 3.6.3 Symptomatic Intracranial Hemorrhage Within 36 h

A significant difference was observed, with more symptomatic intracranial hemorrhage (SICH) within 36 h in the TNK group compared to the standard medical care group (odds ratio = 2.24, 95% CI: 1.04, 4.82; *p* = 0.04, Figure 03(D)). Heterogeneity between the studies was low (*I* ^2^ = 0%). No subgroup analysis was done for this outcome as only three studies reported it (Figure 4C).

#### 3.6.4 SICH Within 48 h

SICH within 48 h were higher in the TNK group compared to the standard medical care group but not enough to gain significant differences between the two treatments (odds ratio = 1.99, 95% CI: 0.77, 5.17; *p* = 0.16, Figure 03(C)). Heterogeneity between the studies was moderate (*I* ^2^ = 32%). No subgroup analysis was done for this outcome as only three studies reported it (Figure 4D).

#### 3.6.5 Any ICH

A significant difference was noted, with more numbers of any ICH found in the TNK group (odds ratio = 1.40, 95% CI: 1.01, 1.94; *p* = 0.04, Figure 03(E)). Heterogeneity between the studies was low (*I* ^2^ = 7%). No subgroup analysis was done for this outcome as only three studies reported it (Figure 4E).

#### 3.6.6 Barthel Index Score >95

Barthel index score was comparable between the TNK group and the standard medical care group. No significant difference was observed between the two treatments (odds ratio = 1.09, 95% CI: 0.72, 1.64; *p* = 0.69, Figure 03(F)) with no heterogeneity between the studies. No subgroup analysis was done for this outcome as only two studies reported it (Figure 4F).

#### 3.6.7 EQ‐5D‐5L (Quality of Life) Score at 90 Days

The EQ‐5D‐5l score between the two groups was found to be comparable with very low mean difference. No significant difference was observed between the two treatments (mean difference = −0.02, 95% CI: −0.21, 0.18; *p* = 0.86, Figure 04(A)). Heterogeneity between the studies was moderate (*I* ^2^ = 60%). No sensitivity and subgroup analysis was done for this outcome as only two studies reported it (Figure 4G).

#### 3.6.8 Change in the NIHSS Score at 7 Days

**FIGURE 5:**
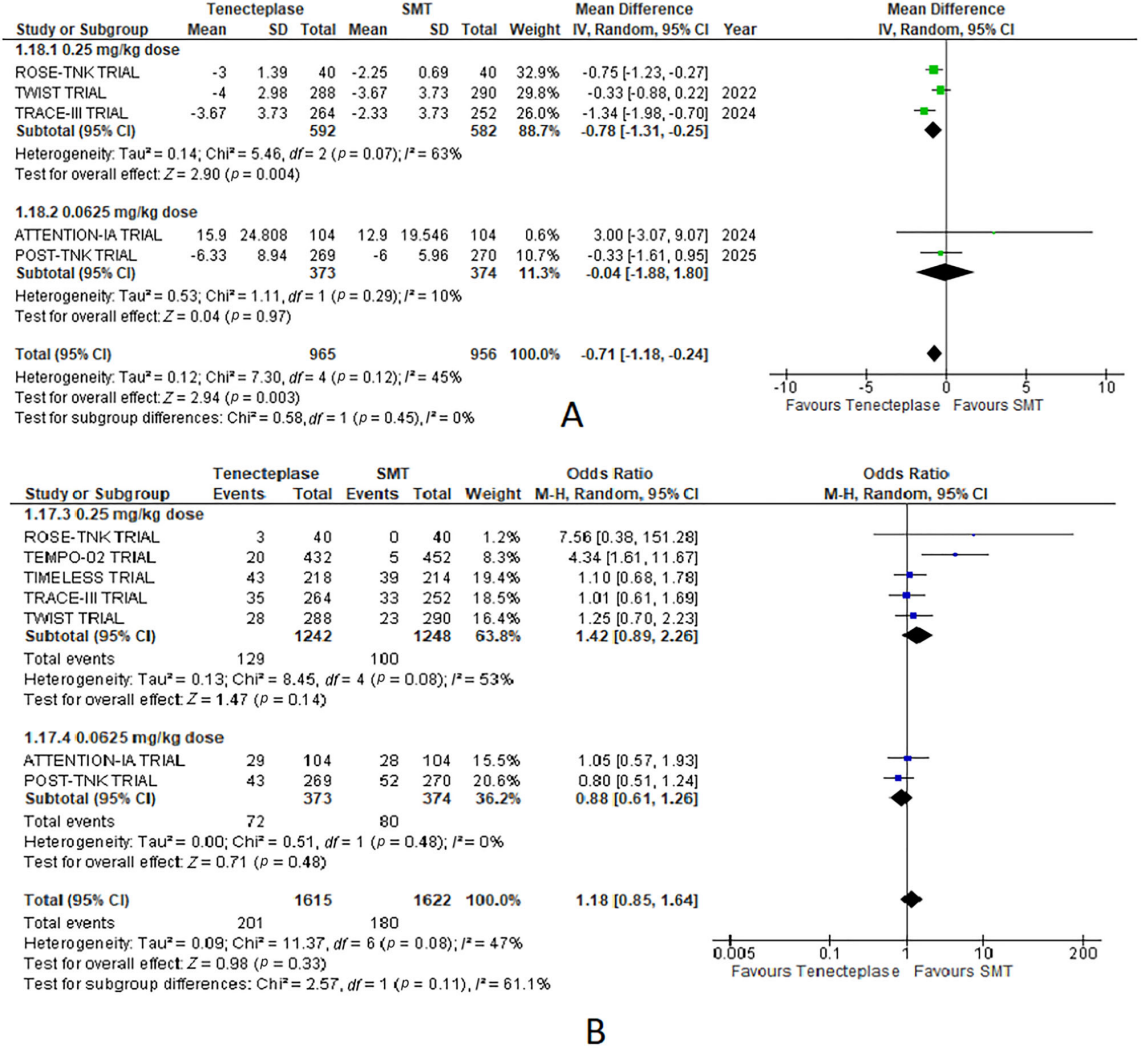
Forest plots for dosed‐based subgroup analysis of outcomes comparing standard medical treatment (SMT): (A) change in the NIHSS score at 7 days and (B) mortality at 90 days.

TNK significantly improved NIHSS scores at 7 days compared to standard medical therapy (mean difference = −0.71, 95% CI: −1.18 to −0.24, *p* = 0.003, Figure 04(B)). Heterogeneity between the studies was moderate (*I* ^2^ = 45%) (Figures 2C and 5A).

#### 3.6.9 Serious Adverse Events

**FIGURE 6:**
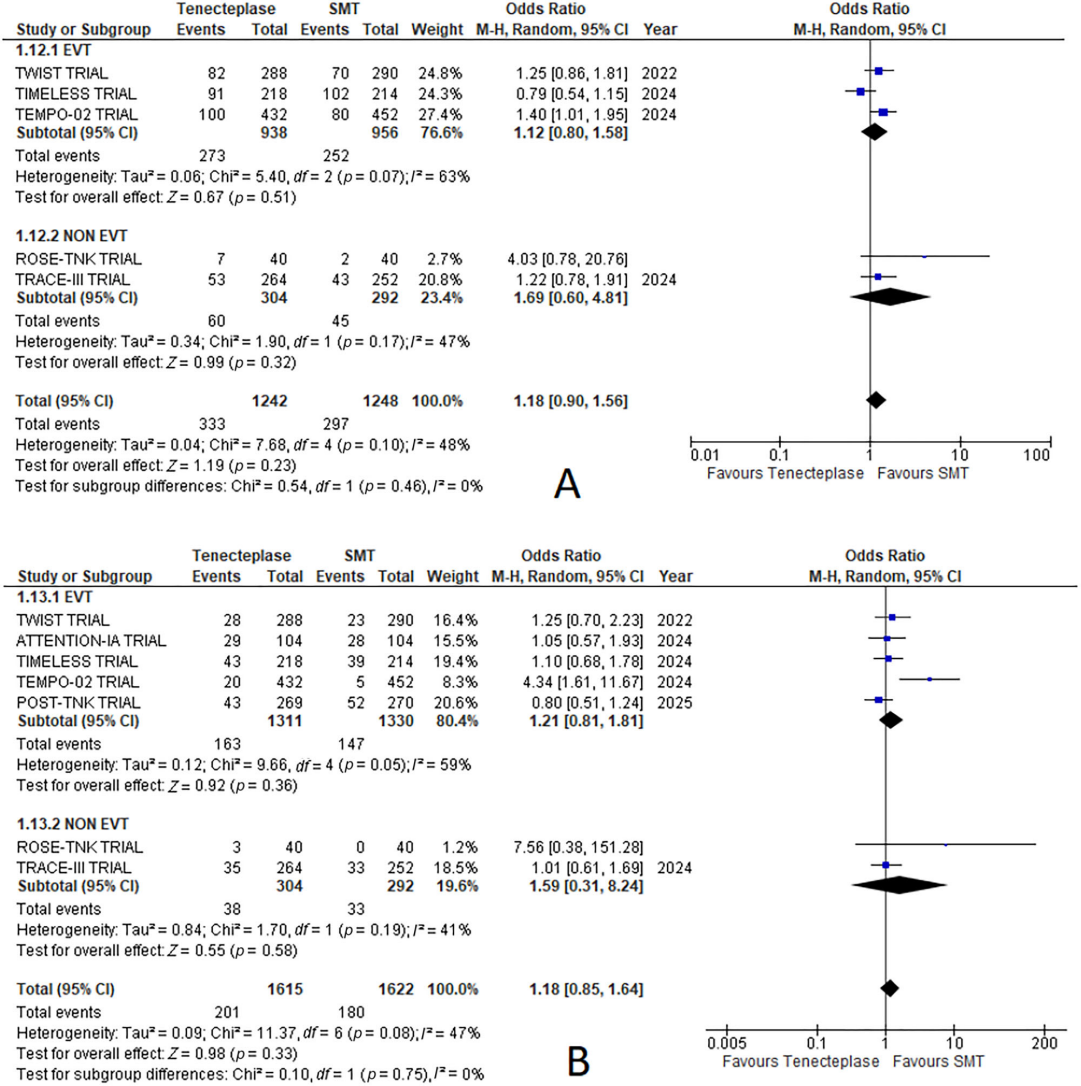
Forest plots of EVT‐based subgroup analysis of outcomes comparing tenecteplase with standard medical treatment (SMT): (A) serious adverse events and (B) mortality at 90 Days.

Serious adverse events were little higher in the TNK group, but not enough to be statistically significant (odds ratio = 1.18, 95% CI: 0.90, 1.56; *p* = 0.23, Figure 04(C)). Heterogeneity between the studies was moderate (*I* ^2^ = 48%) (Figure 6A).

#### 3.6.10 Mortality at 90 Days

Mortality at 90 days was little higher in the TNK group but not enough to gain statistical significance (odds ratio = 1.18, 95% CI: 0.85, 1.64; *p* = 0.33, Figure 04(D)). Heterogeneity between the studies was moderate (*I* ^2^ = 47%) (Figures 6B and 5B).

#### 3.6.11 Stroke Recurrence

The stroke recurrence rate was a little higher in the TNK group compared to the standard medical care group, but this difference did not make the stroke recurrence rate statistically significant between the two groups (odds ratio = 1.23, 95% CI: 0.63, 2.37; *p* = 0.55, Figure 04(E)). No heterogeneity was present between the studies (*I* ^2^ = 0%). No subgroup analysis was done for this outcome as only two studies reported it (Figure 4H).

### 3.7 Subgroup Analysis

In our study, we conducted comprehensive subgroup analyses to evaluate the efficacy and safety of TNK compared to standard medical therapy (SMT) across various outcomes, stratified by dosage and endovascular therapy (EVT) status.​

#### 3.7.1 EVT Status Analysis

##### 3.7.1.1 No Disability (mRS 0–1) at 90 Days

In the EVT subgroup, the odds ratio (OR) was 1.18 (95% CI: 0.99–1.40; *p* = 0.07). The non‐EVT subgroup demonstrated a significant benefit favoring TNK, with an OR of 1.46 (95% CI: 1.02–2.08; *p* = 0.04). Heterogeneity was low for both subgroups (*I* ^2^ = 14% for EVT; *I* ^2^ = 0% for non‐EVT) (Figure 2A).

##### 3.7.1.2 Functional Independence (mRS 0–2) at 90 Days

The EVT subgroup showed an OR of 0.98 (95% CI: 0.81–1.20; *p* = 0.87). In contrast, the non‐EVT subgroup exhibited a significant benefit favoring TNK, with an OR of 1.50 (95% CI: 1.07 to 2.09; *p* = 0.02). Heterogeneity was low for both subgroups (*I* ^2^ = 25% for EVT; *I* ^2^ = 0% for non‐EVT) (Figure 2B).

##### 3.7.1.3 Change in the NIHSS Score at 7 Days

The EVT subgroup showed an MD of −0.31 (95% CI: −0.81 to 0.20; *p* = 0.23). The non‐EVT subgroup demonstrated a significant improvement with TNK, with an MD of −1.00 (95% CI: −1.58 to −0.43; *p* = 0.0006). Heterogeneity was 0% for the EVT group and 52% for the non‐EVT group (Figure 2C).

##### 3.7.1.4 Serious Adverse Events

The EVT subgroup reported an OR of 1.12 (95% CI: 0.80–1.58; *p* = 0.51). The non‐EVT subgroup had an OR of 1.69 (95% CI: 0.60–4.81; *p* = 0.32). Heterogeneity was moderate for both subgroups (*I* ^2^ = 63% for EVT; *I* ^2^ = 47% for non‐EVT) (Figure 6A).

##### 3.7.1.5 Mortality at 90 Days

The EVT subgroup showed an OR of 1.21 (95% CI: 0.81–1.81; *p* = 0.36). The non‐EVT subgroup had an OR of 1.59 (95% CI: 0.31–8.24; *p* = 0.58). Heterogeneity was moderate for both subgroups (*I* ^2^ = 59% for EVT; I: 41%) (Figure 6B).

#### 3.7.2 Dose‐Based Analysis

##### 3.7.2.1 mRS Score at 90 Days

TNK at 0.25 mg/kg yielded an MD of 0.20 (95% CI: −0.03 to 0.43; *p* = 0.09) compared to SMT. Conversely, the 0.0625 mg/kg dose resulted in an MD of −0.65 (95% CI: −1.95 to 0.65; *p* = 0.33). Notably, heterogeneity was low (*I* ^2^ = 0%) for the 0.25 mg/kg subgroup but high (*I* ^2^ = 90%) for the 0.0625 mg/kg subgroup (Figure 3A).

##### 3.7.2.2 No Disability (mRS 0–1) at 90 Days

For the 0.25 mg/kg dose, the OR was 1.21 (95% CI: 0.98–1.49; *p* = 0.08). The 0.0625 mg/kg dose yielded an OR of 1.29 (95% CI: 0.96–1.73; *p* = 0.09). Heterogeneity was low for both subgroups (*I* ^2^ = 31% for 0.25 mg/kg; *I* ^2^ = 0% for 0.0625 mg/kg) (Figure 3B).

##### 3.7.2.3 Functional Independence (mRS 0–2) at 90 Days

The 0.25 mg/kg dose subgroup reported an OR of 1.09 (95% CI: 0.81–1.45; *p* = 0.58), while the 0.0625 mg/kg dose subgroup had an OR of 1.05 (95% CI: 0.79–1.41; *p* = 0.73). Heterogeneity was high for the 0.25 mg/kg subgroup (*I* ^2^ = 60%) and low for the 0.0625 mg/kg subgroup (*I* ^2^ = 0%) (Figure 3C).

##### 3.7.2.4 Change in the NIHSS Score at 7 Days

The 0.25 mg/kg dose subgroup showed a significant improvement (MD: −0.78; 95% CI: −1.31 to −0.25; *p* = 0.004). The 0.0625 mg/kg dose subgroup had an MD of −0.04 (95% CI: −1.88 to 1.80; *p* = 0.97). Heterogeneity was high for the 0.25 mg/kg subgroup (*I* ^2^ = 63%) and low for the 0.0625 mg/kg subgroup (*I* ^2^ = 10%) (Figure 5A ).

##### 3.7.2.5 Mortality at 90 Days

The 0.25 mg/kg dose subgroup reported an OR of 1.42 (95% CI: 0.89–2.26; *p* = 0.14), while the 0.0625 mg/kg dose subgroup had an OR of 0.88 (95% CI: 0.61–1.26; *p* = 0.48). Heterogeneity was moderate for the 0.25 mg/kg subgroup (*I* ^2^ = 53%) and low for the 0.0625 mg/kg subgroup (*I* ^2^ = 0%) (Figure 5B).

### 3.8 Discussion

Our meta‐analysis included seven studies comparing TNK with non‐thrombolytic SMT across multiple clinically relevant outcomes. TNK demonstrated a significant benefit in recanalization at 24 h, higher odds of achieving no disability, and a greater improvement in the NIHSS score at 7 days. However, despite these advantages, no significant differences were observed in key functional outcomes, including the mRS score at 90 days, functional independence, reperfusion at 24 h, quality of life (EQ‐5D‐5L), Barthel index score, and stroke recurrence. Additionally, mortality at 90 days and serious adverse events were not significantly different between the two groups, indicating that TNK did not confer a survival advantage over SMT. Importantly, while TNK was associated with a higher risk of symptomatic intracranial hemorrhage (sICH) within 36 h and an increased likelihood of any ICH, sICH within 48 h showed no significant difference between the two groups. These findings should be viewed in the context of TNK's use in patients for whom standard thrombolysis is not feasible. While thrombolytics outperform medical therapy in eligible patients, our analysis focuses on scenarios where alteplase is contraindicated, unavailable, or delayed beyond standard time windows. This distinction is especially relevant in resource‐limited settings or for late‐presenting patients, where TNK's single‐bolus administration may expand access to thrombolysis.

An important highlight of our analysis was the significant benefit of TNK in achieving no disability (mRS 0–1) at 90 days when compared to SMT. However, no significant differences were observed in median mRS scores or in achieving functional independence (mRS 0–2). These findings closely parallel those of a meta‐analysis by Palaiodimou et al. (2024a), who evaluated TNK (0.25 mg/kg) versus no thrombolysis in an extended 4.5‐ to 24‐h window, reporting similar results—a clear advantage in excellent functional outcome (mRS 0–1), but no significant improvement in mRS 0–2 or overall disability reduction. Unlike Palaiodimou et al. (2024a), who included only trials using the 0.25 mg/kg dose, our study offered a broader dosing perspective, incorporating trials with both 0.25 mg/kg and 0.0625 mg/kg TNK. This broader inclusion enhances the generalizability of our findings. The inclusion of recent landmark trials like ATTENTION‐IA, TEMPO‐2, and POST‐TNK addresses gaps in earlier meta‐analyses and highlights TNK's potential to improve outcomes in situations where alteplase is contraindicated or unavailable. Notably, significant heterogeneity was found in the median mRS at 90 days, which was fully resolved (*I* ^2^ = 0%) through leave‐one‐out analysis excluding the ATTENTION trial—a study that uniquely used the lower 0.0625 mg/kg dose. This suggests that dose variation may account for functional outcome discrepancies, highlighting a possible dose–response relationship favoring the standard 0.25 mg/kg dose. Another distinction lies in the time window of patient inclusion. While Palaiodimou et al. (2024a) focused solely on trials treating patients between 4.5 and 24 h, our analysis also incorporated studies involving patients within 4.5 h of symptom onset, making our findings more inclusive across clinical timeframes. Therefore, our findings support TNK as a practical alternative in populations traditionally excluded from thrombolytic therapy, helping close a long‐standing evidence gap in stroke care.

Despite these differences, both studies reached the same conclusion: TNK improves the chance of excellent recovery (mRS 0–1), but not partial independence (mRS 0–2). Importantly, no significant heterogeneity was observed in mRS 0–1 or mRS 0–2 outcomes in our analysis, reinforcing the consistency and reliability of TNK's efficacy across various subgroups. The favorable outcome in achieving mRS 0–1 may reflect TNK's potent fibrinolytic action and earlier reperfusion, particularly in patients with salvageable penumbra. However, functional independence may be influenced by other factors, such as stroke severity, infarct volume, comorbidities, and access to rehabilitation, which go beyond the scope of thrombolytic intervention alone.

The increased early‐phase risk of sICH with TNK observed in both Palaiodimou et al. (2024a) and our study, significant within 36 h but not at 48 h, is likely due to its prolonged thrombolytic activity, which peaks during the first 24–36 h when ischemic vessels are most vulnerable. This aligns with findings by Yaghi et al. (2015), who noted most sICH events occur between 2 and 10 h post‐thrombolysis with a median time of 8 h. As coagulation pathways recover beyond this window, bleeding risk declines, explaining the lack of later differences. Our more granular time‐point analysis may also explain why we detected increased ICH risk with TNK, unlike Palaiodimou et al. (2024a). Despite early bleeding, mortality remained unchanged, and low heterogeneity across studies—despite varied dosing and treatment windows—supports the consistency and generalizability of TNK's safety profile.

One of the most notable findings of our study was that TNK significantly improved recanalization compared to non‐thrombolytic SMT, but this did not lead to a significant improvement in reperfusion. This highlights a critical distinction—while recanalization reflects vessel reopening, reperfusion involves restoring blood flow to the ischemic brain, and the two are not always synonymous. Factors such as microvascular damage, distal clot embolization, and ischemia‐reperfusion injury can lead to the “no‐reflow” phenomenon, where recanalization fails to result in effective tissue perfusion. This aligns with findings from Mujanovic et al. (2024), who reported that nearly 29% of patients with macrovascular reperfusion still experienced no‐reflow, which was associated with poor functional outcomes. Although meta‐analysis by Kheiri et al. (2018) demonstrated TNK's superiority over alteplase for complete recanalization, its impact on reperfusion remains underexplored. The EXTEND‐IA Part 2 trial found no advantage of 0.4 over 0.25 mg/kg TNK Campbell et al. (2020), and our analysis extended this by including 0.25 and 0.0625 mg/kg doses, again showing no significant benefit on reperfusion or functional outcomes. This suggests reperfusion success may be dose‐independent and influenced more by factors like collaterals, infarct progression, and occlusion characteristics. Notably, our study is the first meta‐analysis to evaluate TNK versus SMT specifically for reperfusion, and we found no significant difference between the two groups.

Our study found no significant difference in serious adverse events (SAEs) or stroke recurrence between TNK and non‐thrombolytic SMT, supporting TNK's favorable safety profile and indicating comparable long‐term vascular risk to conservative treatment. This contrasts with Yao et al.’s network meta‐analysis, which included over 14,000 patients and reported that thrombolytics—including TNK and standard‐dose alteplase—were associated with a higher SAE risk compared to placebo and low‐dose alteplase. However, Yao et al. did not isolate TNK from other agents or doses, making direct comparisons to SMT difficult. Importantly, our analysis primarily assessed TNK at 0.25 mg/kg, a dose believed to offer the best balance between efficacy and safety. The lack of increased SAEs or stroke recurrence at this dose highlights TNK's potential as a safe alternative, especially in patients where alteplase or higher‐dose thrombolysis is not suitable. Since stroke recurrence is largely influenced by secondary prevention, future research should assess whether additional interventions could enhance long‐term outcomes in specific patient subgroups.

Our meta‐analysis found that TNK significantly improved early neurological outcomes, as indicated by a greater change in the NIHSS score at 7 days compared to SMT. However, this early improvement did not translate into significant differences in broader functional outcomes, such as achieving a Barthel index >95 or better EQ‐5D‐5L quality‐of‐life scores. These findings partially align with Rehman et al. (2023), who compared TNK (0.25 mg/kg) to alteplase and observed improved early neurological recovery and excellent functional outcomes at 3 months. A key distinction is that Rehman et al. compared TNK to alteplase, which itself is superior to placebo, while our study compared TNK to non‐thrombolytic SMT, possibly explaining the absence of significant long‐term functional gains. Furthermore, while mRS 0–1 reflects minimal disability, the Barthel index >95 is a stricter criterion for daily independence, which may not be met due to factors such as comorbidities, limited rehabilitation access, or post‐stroke complications. Similarly, the lack of significant EQ‐5D‐5L differences suggests that TNK's impact on long‐term quality of life may be limited by these same factors, despite early clinical improvement.

While the superiority of thrombolysis over SMT is well established in general stroke populations, this relationship has not been clearly validated in patients who are ineligible for EVT—a subgroup that represents a significant proportion of real‐world cases. To address this gap, a recent meta‐analysis by Günkan et al. (2025) focused specifically on patients for whom EVT was not an option, demonstrating that thrombolytic agents, including TNK and alteplase, were associated with significantly better functional outcomes compared to standard medical care. Building on this, our updated meta‐analysis provides a more refined evaluation by isolating the effects of TNK alone in this non‐EVT population. After conducting a subgroup analysis based on EVT status, we observed that TNK significantly improved outcomes in the non‐EVT subgroup, including higher rates of achieving no disability (mRS 0–1), greater functional independence (mRS 0–2), and better NIHSS score improvements. These benefits may be attributed to TNK's ease of administration and fibrin‐specific action, making it particularly advantageous in settings where mechanical reperfusion is not pursued. Safety outcomes, such as serious adverse events and 90‐day mortality, were consistent across both EVT and non‐EVT groups, suggesting that TNK's safety profile remains stable regardless of EVT use. This indicates its potential as a standalone reperfusion strategy in environments lacking immediate EVT access.

Additionally, subgroup analysis based on TNK dosing revealed no significant differences between the 0.25 and 0.0625 mg/kg doses across most outcomes. However, the 0.25 mg/kg dose demonstrated a significant improvement in NIHSS scores at 7 days, suggesting a dose‐dependent effect on early neurological recovery. This aligns with prior studies indicating that the 0.25 mg/kg dose may enhance early reperfusion and clinical improvement (Parsons et al. 2012). Nonetheless, given the absence of significant differences in other key functional outcomes, the overall advantage of a higher dose remains uncertain. These findings suggest that while the 0.25 mg/kg dose of TNK may offer superior early neurological improvement, further studies are needed to determine whether this translates into better long‐term functional recovery and to evaluate its safety profile.

### 3.9 Strengths and Limitations

Our meta‐analysis stands out as the most comprehensive and up‐to‐date synthesis comparing TNK with SMT without thrombolytics, offering valuable insights into its efficacy and safety. Unlike previous meta‐analyses that focused narrowly on comparisons between TNK and alteplase or that restricted inclusion to specific time windows or fixed dosages, our study did not limit trials based on treatment window or TNK dose. This inclusive approach allowed us to capture a broader spectrum of real‐world clinical scenarios, including trials using both 0.25 and 0.0625 mg/kg doses of TNK, as well as patients presenting both early and late after stroke onset. A major strength of our study is the breadth and depth of outcomes assessed, making it the largest and most exhaustive analysis to date on this topic. We evaluated a wide range of endpoints providing a well‐rounded picture of TNK's performance.

However, certain limitations must be acknowledged. Significant heterogeneity was observed in outcomes such as mean mRS score at 90 days, recanalization, reperfusion, and EQ‐5D‐5L scores. While heterogeneity in mRS score was mitigated through leave‐one‐out sensitivity analysis, limited reporting across studies precluded similar analysis for other outcomes, which were often reported by only one or two trials. Further exploration of heterogeneity through meta‐regression or subgroup analyses was also not feasible for other outcomes due to the limited number of contributing studies. As many of these outcomes were reported by only one or two trials, making such analyses statistically underpowered and potentially misleading. The small number of studies with limited patient population and variability in trial design, inclusion criteria, imaging protocols, thrombus characteristics, and treatment windows likely contributed to these inconsistencies. The inclusion of trials spanning both early and extended time frames further adds to this variability. Differences in imaging protocols or stroke severity across studies may have also influenced the observed heterogeneity. The lack of patient‐level data limited more detailed analyses, and inconsistencies in functional outcome definitions across studies posed challenges in result synthesis. Additionally, potential publication bias cannot be excluded given the limited number of available studies. Another limitation is the lack of long‐term follow‐up data and the absence of a direct head‐to‐head comparison with alteplase, which restricts definitive conclusions about TNK's relative efficacy. Nonetheless, by broadening the inclusion criteria and systematically analyzing both efficacy and safety across diverse clinical contexts, our meta‐analysis fills a critical gap in the literature and underscores the need for future large‐scale trials evaluating TNK in non‐thrombolytic treatment settings and extended windows.

## Author Contributions

**Zaryab Bacha**: conceptualization, methodology, data curation, investigation, writing–original draft, writing–review and editing. **Javeria Javed**: writing–review and editing, project administration, writing–original draft, resources, formal analysis, conceptualization. **Maheen Sheraz**: writing–review and editing, writing–original draft, project administration, visualization, data curation, investigation. **Muhammad Abdullah Ali**: methodology, software, supervision, formal analysis, writing–original draft, writing–review and editing. **M. Ehtisham Wali Khan**: writing–review and editing, writing–original draft, visualization, formal analysis, data curation, investigation. **Sufyan Shahid**: formal analysis, validation, resources, supervision, writing–original draft, writing–review and editing. **Asad Iqbal**: writing–review and editing, writing–original draft, visualization, formal analysis, validation, project administration. **Mahnoor Qasim**: Writing–original draft, writing–review and editing, resources, visualization, validation, formal analysis. **Abdullah Afridi**: Methodology, data curation, writing–original draft, writing–review and editing, visualization, supervision. **Fazia Khattak**: Writing–review and editing, writing–original draft, visualization, formal analysis, validation, investigation. **fathimathul Henna**: Writing–review and editing, writing–original draft, methodology, data curation, investigation, validation. **Mazhar Ali Shah**: Writing–review and editing, writing–original draft, methodology, data curation, validation, formal analysis. **Raheel Ahmed**: Writing–original draft, writing–review and editing, resources, formal analysis, validation, investigation.

## Conflicts of Interest

The authors declare no conflicts of interest.

## Peer Review

The peer review history for this article is available at https://publons.com/publon/10.1002/brb3.70791.

## Supporting information




**Supporting Material**: brb370791‐sup‐0001‐SuppMat.docx

## Data Availability

All data used are cited in the manuscript.
