# Supplementary material for: Efficacy and Safety of Tenecteplase in Acute Ischemic Stroke: A Meta‐Analysis of Randomized Controlled Trials
Source: Brain Behav. 2025 Sep 1;15(9):e70791. doi: 10.1002/brb3.70791 (PMC12402402; doi:10.1002/brb3.70791)
Supplement: Supplementary file 1 — Supporting Material: brb370791‐sup‐0001‐SuppMat.docx [file BRB3-15-e70791-s001.docx]

| **PUBMED(260)**  ("Tenecteplase"[Mesh] OR "Metalyse" OR "TNKase") AND ("Ischemic Stroke"[Mesh] OR "Ischemic Strokes" OR "Stroke, Ischemic" OR "Ischaemic Stroke" OR "Ischaemic Strokes" OR "Stroke, Ischaemic" OR "Acute Ischemic Stroke" OR "Acute Ischemic Strokes" OR "Ischemic Stroke, Acute" OR "Stroke, Acute Ischemic" OR "Cryptogenic Ischemic Stroke" OR "Cryptogenic Ischemic Strokes" OR "Ischemic Stroke, Cryptogenic" OR "Stroke, Cryptogenic Ischemic" OR "Cryptogenic Embolism Stroke" OR "Cryptogenic Embolism Strokes" OR "Embolism Stroke, Cryptogenic" OR "Stroke, Cryptogenic Embolism" OR "Cryptogenic Stroke" OR "Cryptogenic Strokes" OR "Stroke, Cryptogenic" OR "Wake-up Stroke" OR "Stroke, Wake-up" OR "Wake up Stroke" OR "Wake-up Strokes") |
| --- |
| **EMBASE**  ('tenecteplase'/exp OR 'Metalyse' OR 'TNKase') AND ('ischemic stroke'/exp OR 'ischemic strokes' OR 'stroke, ischemic' OR 'ischaemic stroke' OR 'ischaemic strokes' OR 'stroke, ischaemic' OR 'acute ischemic stroke' OR 'acute ischemic strokes' OR 'ischemic stroke, acute' OR 'stroke, acute ischemic' OR 'cryptogenic ischemic stroke' OR 'cryptogenic ischemic strokes' OR 'ischemic stroke, cryptogenic' OR 'stroke, cryptogenic ischemic' OR 'cryptogenic embolism stroke' OR 'cryptogenic embolism strokes' OR 'embolism stroke, cryptogenic' OR 'stroke, cryptogenic embolism' OR 'cryptogenic stroke' OR 'cryptogenic strokes' OR 'stroke, cryptogenic' OR 'wake-up stroke' OR 'stroke, wake-up' OR 'wake up stroke' OR 'wake-up strokes') |
| **WEB OF SCIENCE**  TS=("Tenecteplase" OR "Metalyse" OR "TNKase") AND TS=("Ischemic Stroke" OR "Ischemic Strokes" OR "Stroke, Ischemic" OR "Ischaemic Stroke" OR "Ischaemic Strokes" OR "Stroke, Ischaemic" OR "Acute Ischemic Stroke" OR "Acute Ischemic Strokes" OR "Ischemic Stroke, Acute" OR "Stroke, Acute Ischemic" OR "Cryptogenic Ischemic Stroke" OR "Cryptogenic Ischemic Strokes" OR "Ischemic Stroke, Cryptogenic" OR "Stroke, Cryptogenic Ischemic" OR "Cryptogenic Embolism Stroke" OR "Cryptogenic Embolism Strokes" OR "Embolism Stroke, Cryptogenic" OR "Stroke, Cryptogenic Embolism" OR "Cryptogenic Stroke" OR "Cryptogenic Strokes" OR "Stroke, Cryptogenic" OR "Wake-up Stroke" OR "Stroke, Wake-up" OR "Wake up Stroke" OR "Wake-up Strokes") |

Supplementary Table 1. Search strategy

| **Summary of findings:** | | | | | | |
| --- | --- | --- | --- | --- | --- | --- |
| **Tenecteplase compared to Standard medical care in ischemic stroke** | | | | | | |
| **Patient or population:** ischemic stroke  **Setting:**  **Intervention:** Tenecteplase  **Comparison:** Standard medical care | | | | | | |
| Outcomes | **Anticipated absolute effects^*^** (95% CI) | | Relative effect (95% CI) | № of participants (studies) | Certainty of the evidence (GRADE) | Comments |
|  | **Risk with Standard medical care** | **Risk with Tenecteplase** |  |  |  |  |
| Mean mRS score at 90 days assessed with: MD | The mean mean mRS score at 90 days was **0** | **0** (0 to 0 ) | - | 2089 (4 RCTs) | ⨁⨁⨁◯ Moderate^a^ |  |
| Excellent functional outcomes (mRS 0-1 ) at 90 days (Subgroup EVT/ NON EVT) assessed with: OR | 440 per 1,000 | **489 per 1,000** (450 to 527) | **OR 1.22** (1.04 to 1.42) | 3260 (7 RCTs) | ⨁⨁⨁⨁ High |  |
| Good functional outcomes (mRS 0-2) at 90 days (EVT/NON EVT subgroups) assessed with: OR | 593 per 1,000 | **609 per 1,000** (559 to 656) | **OR 1.07** (0.87 to 1.31) | 3260 (7 RCTs) | ⨁⨁⨁◯ Moderate^a^ |  |
| Recanilization at 24 hour assessed with: OR | 309 per 1,000 | **595 per 1,000** (316 to 824) | **OR 3.28** (1.03 to 10.44) | 903 (2 RCTs) | ⨁⨁◯◯ Low^b,c^ |  |
| Reperfusion (T1 C1 2b-3) at 24 hours assessed with: OR | 321 per 1,000 | **386 per 1,000** (246 to 546) | **OR 1.33** (0.69 to 2.54) | 824 (2 RCTs) | ⨁⨁◯◯ Low^a,b^ |  |
| Symptomatic intracranial hemorrhage (SiCH) within 36 hours assessed with: OR | 18 per 1,000 | **38 per 1,000** (18 to 79) | **OR 2.24** (1.04 to 4.82) | 1156 (3 RCTs) | ⨁⨁⨁⨁ High |  |
| Symptomatic intracranial hemorrhage (SiCH) within 48 hours assessed with: OR | 18 per 1,000 | **36 per 1,000** (14 to 88) | **OR 1.99** (0.77 to 5.17) | 1503 (3 RCTs) | ⨁⨁⨁◯ Moderate^a^ |  |
| Any intracranial hemorrhage  assessed with: OR | 173 per 1,000 | **227 per 1,000** (174 to 289) | **OR 1.40** (1.01 to 1.94) | 1197 (3 RCTs) | ⨁⨁⨁⨁ High |  |
| Barthel Index (BI) score assessed with: OR | 472 per 1,000 | **493 per 1,000** (391 to 594) | **OR 1.09** (0.72 to 1.64) | 378 (2 RCTs) | ⨁⨁⨁◯ Moderate |  |
| Quality of life (EQ-5D-5L) at 90 days assessed with: MD | The mean quality of life (EQ-5D-5L) at 90 days was **0** | **0** (0 to 0 ) | - | 747 (2 RCTs) | ⨁⨁◯◯ Low^a,b^ |  |
| Change in NIHSS score at 7 days assessed with: MD | The mean change in NIHSS score at 7 days was **0** | **0** (0 to 0 ) | - | 1921 (5 RCTs) | ⨁⨁⨁⨁ High |  |
| Serious adverse events assessed with: OR | 238 per 1,000 | **269 per 1,000** (219 to 328) | **OR 1.18** (0.90 to 1.56) | 2490 (5 RCTs) | ⨁⨁⨁◯ Moderate^a^ |  |
| Mortality at 90 days assessed with: OR | 111 per 1,000 | **128 per 1,000** (96 to 170) | **OR 1.18** (0.85 to 1.64) | 3237 (7 RCTs) | ⨁⨁⨁◯ Moderate^a^ |  |
| Stroke recurrance  assessed with: OR | 23 per 1,000 | **28 per 1,000** (15 to 53) | **OR 1.23** (0.63 to 2.37) | 1462 (2 RCTs) | ⨁⨁⨁◯ Moderate^a^ |  |
| Excellent functional outcomes at 90 days (subgroups on dose basis) (mRS 0-1) assessed with: OR | 440 per 1,000 | **489 per 1,000** (450 to 527) | **OR 1.22** (1.04 to 1.42) | 3260 (7 RCTs) | ⨁⨁⨁⨁ High |  |
| Good functional outcomes at 90 days (subgroups on dose basis) assessed with: OR | 593 per 1,000 | **609 per 1,000** (559 to 656) | **OR 1.07** (0.87 to 1.31) | 3260 (7 RCTs) | ⨁⨁⨁◯ Moderate^a^ |  |
| Mortality at 90 days (subgroups on dose basis) assessed with: OR | 111 per 1,000 | **128 per 1,000** (96 to 170) | **OR 1.18** (0.85 to 1.64) | 3237 (7 RCTs) | ⨁⨁⨁◯ Moderate^a^ |  |
| Change in NIHSS score at 7 days (subgroups on dose basis) assessed with: MD | The mean change in NIHSS score at 7 days (subgroups on dose basis) was **0** | **0** (0 to 0 ) | - | 1921 (5 RCTs) | ⨁⨁⨁⨁ High |  |
| ***The risk in the intervention group** (and its 95% confidence interval) is based on the assumed risk in the comparison group and the **relative effect** of the intervention (and its 95% CI).  **CI:** confidence interval; **OR:** odds ratio | | | | | | |
| **GRADE Working Group grades of evidence High certainty:** we are very confident that the true effect lies close to that of the estimate of the effect. **Moderate certainty:** we are moderately confident in the effect estimate: the true effect is likely to be close to the estimate of the effect, but there is a possibility that it is substantially different. **Low certainty:** our confidence in the effect estimate is limited: the true effect may be substantially different from the estimate of the effect. **Very low certainty:** we have very little confidence in the effect estimate: the true effect is likely to be substantially different from the estimate of effect. | | | | | | |

Supplementary Table 2. GRADE assessment to assess the certainty of evidence.


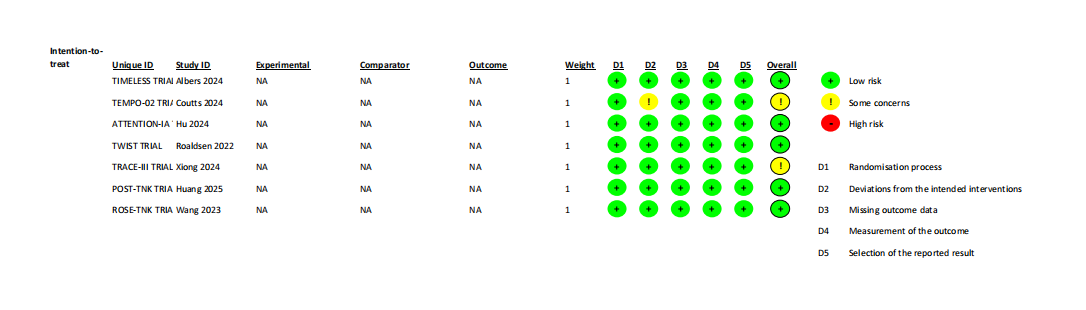
Supplementary Figure 1. Quality assessment of included studies


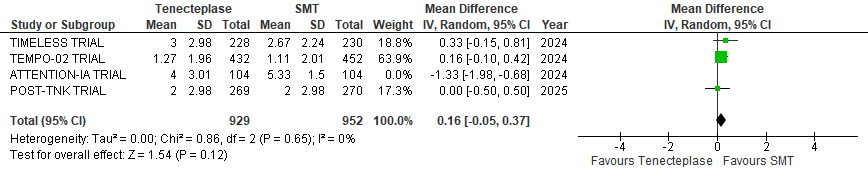
Supplementary Figure 2. Forest plot of Mean mRS score at 90 days after performing sensitivity

analysis.
